# Effect of lumbopelvic control on landing mechanics and lower extremity muscles’ activities in female professional athletes: implications for injury prevention

**DOI:** 10.1186/s13102-021-00331-y

**Published:** 2021-08-29

**Authors:** Paria Fadari Dehcheshmeh, Farzaneh Gandomi, Nicola Maffulli

**Affiliations:** 1 Department of Sport Injuries and Corrective Exercises, Sports Sciences Faculty, Razi University, Kermanshah, Iran; 2 Department of Musculoskeletal Disorders, Via Salvador Allende, 43, 84081 Baronissi, Italy; 3 Clinica Ortopedica, Ospedale San Giovanni di Dio e Ruggi D’Aragona, Largo Città di Ippocrate, 84131 Salerno, Italy; 4 Barts and the London School of Medicine and Dentistry, Centre for Sports and Exercise Medicine, Mile End Hospital, Queen Mary University of London, 275 Bancroft Road, London, E14DG England; 5 School of Pharmacy and Bioengineering, Faculty of Medicine, Guy Hilton Research Centre, Keele University, Thornburrow Drive, Hartshill, Stoke-on-Trent, ST4 7QB England

**Keywords:** Lumbopelvic control, Landing mechanics, Lower limb injury, Jump-landing

## Abstract

**Background:**

Lumbopelvic control (LPC) has recently been associated with function, kinesiology, and load distribution on the limb. However, poor LPC has not been studied as a risk factor for lower limb injury in sports requiring frequent jump landings. The present study investigated the effects of LPC on landing mechanics and lower limb muscle activity in professional athletes engaged in sport requiring frequent landing.

**Methods:**

This study was conducted on 34 professional female athletes aged 18.29 ± 3.29 years with the height and body mass of 173.5 ± 7.23 cm and 66.79 ± 13.37 kg, respectively. The landing error scoring system (LESS) and ImageJ software were used to assess landing mechanics. Wireless electromyography was also used to record the activity of the gluteus medius (GMed), rectus femoris, and semitendinosus. Lumbopelvic control was evaluated using the knee lift abdominal test, bent knee fall-out, active straight leg raising, and the PRONE test using a pressure biofeedback unit. Based on the LPC tests results, the participants were divided into two groups of proper LPC (n = 17) and poor LPC (n = 17).

**Results:**

There were significant differences between the groups with proper and poor LPC in terms of the LESS test scores (*P* = 0.0001), lateral trunk flexion (*P* = 0.0001), knee abduction (*P* = 0.0001), knee flexion (*P* = 0.001), trunk flexion (*P* = 0.01), and GMed muscle activity (*P* = 0.03). There were no significant differences in the activity of the rectus femoris and semitendinosus muscles, and ankle dorsiflexion (*P* > 0.05).

**Conclusions:**

Poor lumbopelvic control affects the kinematics and activity of the lower limb muscles, and may be a risk factor for lower limb injuries, especially of the knee.

## Background

The recognition of risk factors and injury mechanisms is essential to the successful prevention of injuries [1]. Injuries of the lower limbs (especially the knee) are common in athletes, with long term consequences, such as premature osteoarthritis and poor performance [2]. Frequent jumps and landings impose extremely high loads on the lower limbs in sports such as basketball, volleyball, and handball [3].

In the sports involving frequent jump-landing skills, the lower limb is injured in approximately 60% of the cases [4]: 45–86% of acute knee and ankle injuries in basketball and volleyball [5] and 70% of anterior cruciate ligament (ACL) ruptures in handball players [6] occur after a landing. The most important risk factors for these injuries include excessive knee valgus, lateral movements of the trunk, poor pelvic stability [3, 7], and landing from a jump, which is a common sports manoeuvre and is reported in non-contact ACL injuries [8]. Additionally, the kinematic of the trunk while landing is an important contributing factor to ACL injury [9].

The National Institute of Sports Rehabilitation has recommended strength exercises, stabilization, and control of the lumbo-pelvic-hip complex in injury prevention programs [10]. Furthermore, core stability in lower limb function has been reported to be effective in this regard, with weakness in this area a predictor of the occurrence of lower limb injuries [11].

Poor core stability, which refers to musculature control around the lumbopelvic region in order to maintain functional stability in a neutral position, thereby assisting the generation and transfer of energy from the trunk to the extremities, is a risk factor for lower limb injuries [12]. However, only the isometric strength of the trunk and hip muscles has been evaluated in studies on the effects of core stability training on the occurrence of lower extremities injuries [12–14]. In addition to the strength and endurance of the lumbopelvic-hip complex, optimal neuromuscular control is essential to the prevention of uncontrolled and compensatory movements [10].

Recently, lumbopelvic control (LPC) has been identified as a risk factor for kinematic disruptions and upper limb injuries [13]. LPC is defined as the ability to move or stabilize the lumbopelvic region in response to internal or external perturbations [13], and depends on the integrity of the passive structures and appropriate dynamic neuromuscular control [15]. Therefore, poor neuromuscular control of the lumbopelvic-hip complex, which leads to trunk muscle imbalance, synergist dominance, and compensatory movements, could be considered an important risk factor for lower extremity injuries in athletes [16], which is intensified when occurring in high-risk skills, such as landing. For instance, single-leg landings with the trunk in flexion could reduce the risk of ACL injury compared to when the trunk is in an upright position [17]. Poor LPC may affect the muscle activity of the gluteus medius, rectus femoris, and hamstrings, as they insert or originate on the pelvis, thereby playing a role in knee and lower limb injuries [3, 18]. Moreover, one-leg stance conditions are often integrated into fall- and sports-related injury risk assessment protocols [19]. Therefore, it is essential to evaluate the activity of these muscles while standing on one leg.

Some authors have suggested an association between the lumbopelvic-hip complex and lower extremity injuries [20, 21]. For instance, Burnham et al. reported that hip and trunk muscle function is positively correlated with single-leg step-down performance [18]. Furthermore, Leetun et al. [14] emphasized the importance of proximal stabilization for the prevention of lower extremity injuries. Trunk displacement and lateral displacement are deficits in the neuromuscular control of the trunk, as well as the strongest predictors of knee ligament injury [22]. Therefore, evaluation of LPC as a risk factor for lower limb injuries seems essential.

Various methods are used to assess LPC. In the present study, and based on the findings of Roussel et al. [23], multidimensional lumbopelvic movements were evaluated using a biofeedback pressure device. In the mentioned study, the authors assessed the ability for the control and reposition of the lumbopelvic complex when challenged in different directions [23]. Despite the high prevalence of lower limb injuries (especially knee injuries) among athletes engaged in sports requiring frequent landings, the main causes of these injuries remain unknown, and no studies have investigated the effects of poor LPC on the landing mechanics of professional athletes. We hypothesized that:

- 1. The landing mechanics differ between athletes with proper LPC and poor LPC.
- 2. The activity of the muscles acting on the knee would differ between athletes with proper LPC and poor LPC.

## Methods

### Participants

A total of thirty-four female professional basketball, volleyball, and handball players (mean age: 18.29 ± 3.29 years; mean height: 173.5 ± 7.23 cm; mean body mass: 66.79 ± 13.37 kg) playing in the Iranian Pro League and Second Division volunteered to participate in the study in two groups of proper LPC and poor LPC (n = 17). The objectives and procedures were explained to the participants, and written informed consent was obtained in accordance with the Declaration of Helsinki. The research protocol was approved by the Ethics Committee of Razi University of Iran (code: IR.RAZI.REC.1399.007). The study was performed during September 10-November 6, 2020 at Razi University Sports Rehabilitation Laboratory.

**Fig. 1:**
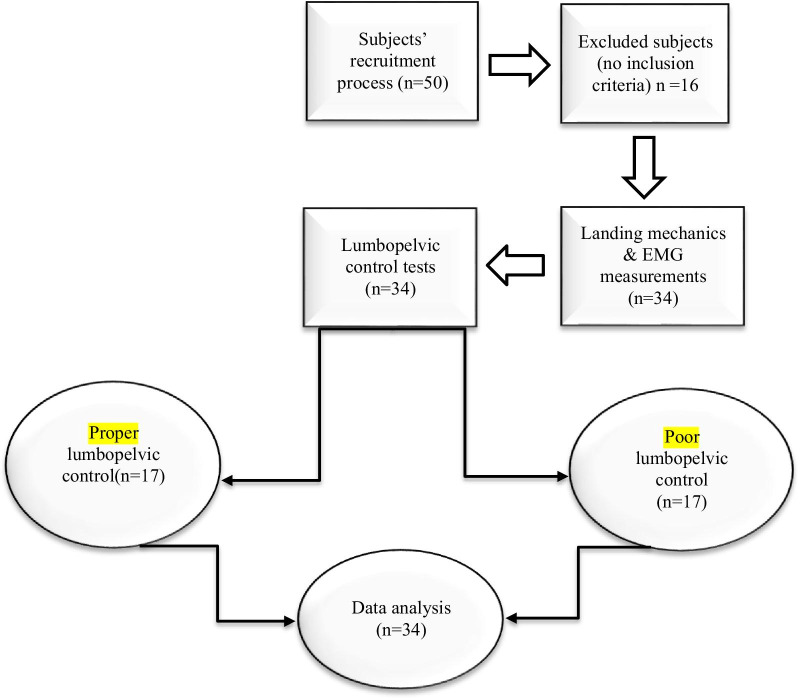
Study flow chart

The exclusion criteria of the study were the age of less than 17 years or more than 25 years, history of lower back injury or severe lower extremity injuries affecting the normal lower extremity function of the athletes, and neuromuscular disorders (Fig. 1). Since the present study was performed during the COVID-19 pandemic, the subjects were initially referred to the university health centre and excluded if diagnosed with abnormal symptoms, such as high body temperature (> 37) or hypoxia (< 93%). After each subject left the laboratory, the environment was completely disinfected with 70% alcohol.

### Procedures

A case–control design was used to assess the differences in the landing mechanic and lower extremity muscles activity between the athletes with proper and poor LPC. Data were collected on the age, weight, height, dominant lower limb, number of the years in high-performance sports, and duration of weekly training (hour). Prior to each assessment, the subjects were asked to warm-up for 10 min by walking, running, and jumping. Two cameras (frontal-sagittal plane views; model: Canon Eos 80D EFS 18–135 mm f/3.5–5.6 IS USM Kit Digital Camera), the landing error scoring system (LESS), and the ImageJ software were used to assess landing mechanics. wireless electromyography (EMG) (Noraxon, Scottsdale, AZ 85260) was also recorded based on the activity of the gluteus medius (GMed), rectus femoris (RF), and semitendinosus (ST). Finally, Lumbopelvic control was evaluated using the knee lift abdominal test (KLAT), bent knee fall-out (BKFO), active straight leg raising (ASLR), and PRONE test by the pressure biofeedback unit (PBU) (Stabilizer®, Chattanooga Group Inc., Hixson, TN, USA). Each test was performed in triplicate, and the mean values obtained in the evaluations were analyzed. In order to prevent bias, the LPC tests were performed at the end of other evaluations, and none of the study subjects were provided with feedback or training on proper landing.

#### Landing mechanics

In this study, jump-landing tasks were simultaneously recorded by two standard cameras. The cameras were mounted on tripods placed 3.5 m in front of and to the side of the dominant leg of the landing area at the lens-to-floor distance of 1.3 m. The markers were placed on the anterior superior iliac spine (ASIS) bilaterally, center of the patella, and center of the talocrural joint in the frontal plane, and some markers were placed on the dominant side greater trochanter, acromion, lateral femoral epicondyle, fifth metatarsal, and lateral malleolus in the sagittal plane for 2D kinematic analysis.

**Fig. 2:**
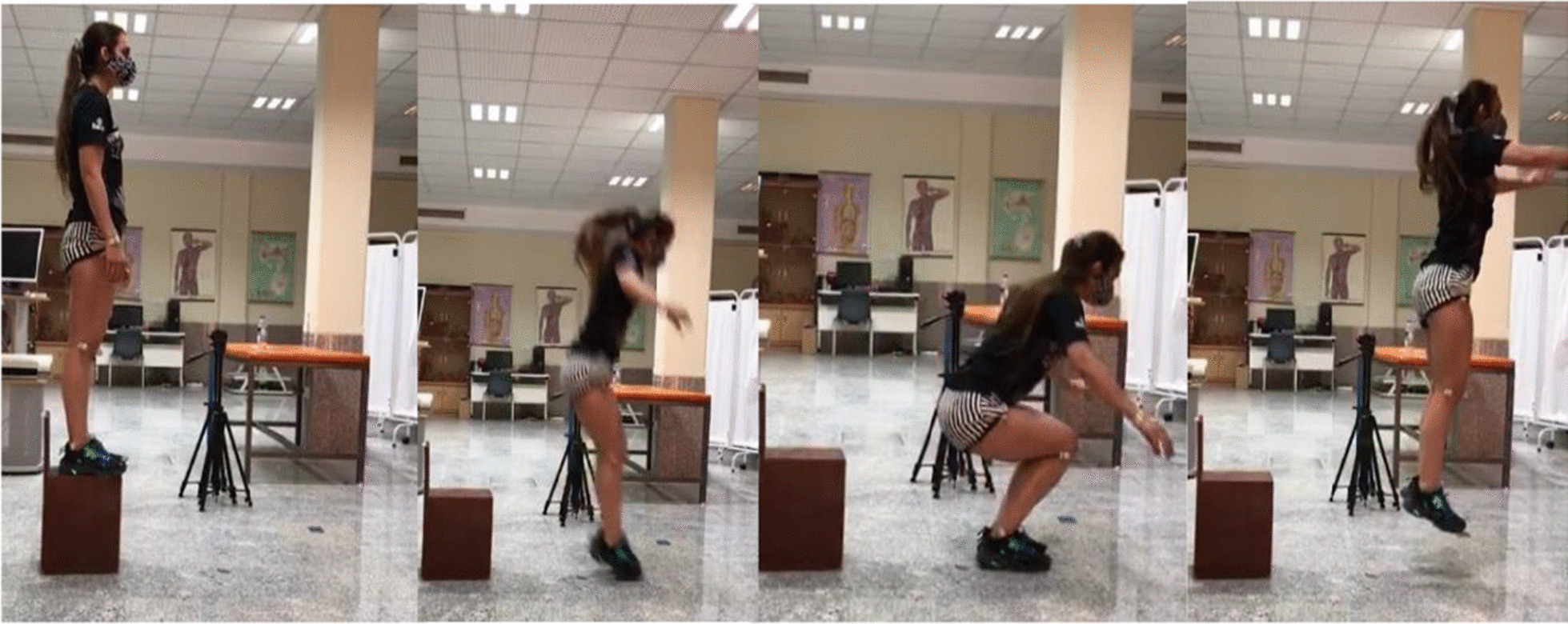
Landing error scoring system demonstration

The LESS is used to evaluate the landing technique of an individual based on 17 easily observable criteria. The participants were asked to jump forward from a 30-cm-tall box, land on a marked spot on the ground that was half their height away from the box, and immediately jump vertically as high as possible (Fig. 2). At this stage, the score of the subjects was calculated out of a total of 17, and was inversely proportional to the LESS performance. The scoring criteria and description of the LESS have been previously reported [24]. The LESS provides a valid, two-dimensional assessment of lower extremity and trunk kinematics and has excellent intraclass correlation-coefficient (ICC) (SEM = 0.42) and good interrater reliability ICC (SEM = 0.71) [25]. The researcher replayed the videos using the Kinovea software version 0.8.15 (www.kinovea.org) and scored the three trials using the 17-item LESS scoring sheet.

ImageJ software was used to evaluate the landing mechanics at the contact time. Knee flexion, trunk flexion, and ankle dorsiflexion in the sagittal plane and trunk inclination and knee abduction in the frontal plane were measured at the contact time in the jump-landing task for each participant. The examiner separated the frontal and sagittal plane contact time frames using the Kinovea software, transferring them to the ImageJ software to accurately measure the angles. In the sagittal plane, trunk flexion was measured as the angle between the line formed by the centre of the shoulder joint and the greater trochanter [26]. Knee flexion was also measured as the angle between the greater trochanter and lateral malleolus with the lateral knee joint serving as the fulcrum. Ankle dorsiflexion was measured as the angle between a line from the lateral knee joint through the lateral malleolus and a line parallel with the fifth metatarsal. In the frontal plane, the lateral trunk movement angle was considered as the angle between a vertical line starting at the ipsilateral anterior superior iliac spin (ASIS) and a line between the ipsilateral ASIS and the manubrium of the sternum [26]. The knee abduction angle was also defined as the angle between the line formed by the ASIS, the midpoint of the medial and lateral epicondyles of the femur, the line formed by the midpoint of the medial and lateral epicondyles of the femur, and the midpoint of the medial and lateral malleoli. The jump-landing skill was assessed in triplicate in each subject, and the mean angles were calculated and analysed [27]. Notably, none of the participants were instructed on proper landing techniques.

#### Electromyography of muscles (EMG)

Wireless surface EMG electrodes were oriented parallel to the muscle fibres and placed on the GMed, RF, and ST muscles of the dominant leg in accordance with the SENIAM recommendations. The GMed was positioned 50% on the line from the iliac crest to the trochanter, RF was placed at 50% of the distance on a straight line between the ASIS and the proximal patellar margin, and ST placed at 50% of the distance on a straight between the ischial tuberosity and medial epicondyle [28].

After shaving and cleaning the sites with 70% alcohol to reduce skin impedance, gel-type Ag/AgCl electrodes (diameter: 20 mm; Skintact, Austria) were attached to the muscles belly of the dominant leg. The surface EMGs were amplified 500 times, sampled at 1500 Hz, and digitized using a 16-bit analog to a digital converter. The signals were filtered at the bandwidth of 10–500 Hz to eliminate the noise recorded during data collection. The mean time (30 s) was calculated during the test [26]. The subjects performed 30 s of a one-leg stance task by the dominant leg. To do so, the subjects were asked to stay with their feet positioned on a specific tape on the floor and maintain the posture for 30 s. The timer was started once the subject had established their balance. If the subject lost their balance during the task (or moved their feet from the tape), the trial was terminated and restarted until they were able to stay balanced for throughout the entire 30-s trial.

The EMG data were normalized to maximum voluntary isometric contraction (MVIC). Maximum voluntary isometric contractions were carried out for each muscle. For the GMed, the subjects lay on their non-dominant side and abducted their dominant leg against resistance. For RF, the subjects sat upright with their knees flexed at a 90° angle, with the ankle of the dominant leg restrained from extending, and attempted to extend their knee. For ST, the subjects were in the same position, and the ankle of the dominant leg was restrained from flexing, with the subject attempting to flex their knee. MVCs were also performed for 3–5 s three times for each muscle with a 10-s rest interval between the efforts. Verbal encouragement was provided throughout these tests [28].

#### LPC tests

At this stage, four LPC tests were performed on the subjects (KLAT, BKFO, ASLR, and PRONE) by PBU in a random order in order to avoid order effects. A manual chronometer (BAT CR2032, China) was used to identify the duration (second) that the subjects maintained each position in the ASLR and PRONE tests. Moreover, a simple long arm goniometer (Goniometer set, Gamatpoyan, Iran) with a 360° angle was used to control the starting positions of the hips and knees during KLAT and BKFO.

**Fig. 3:**
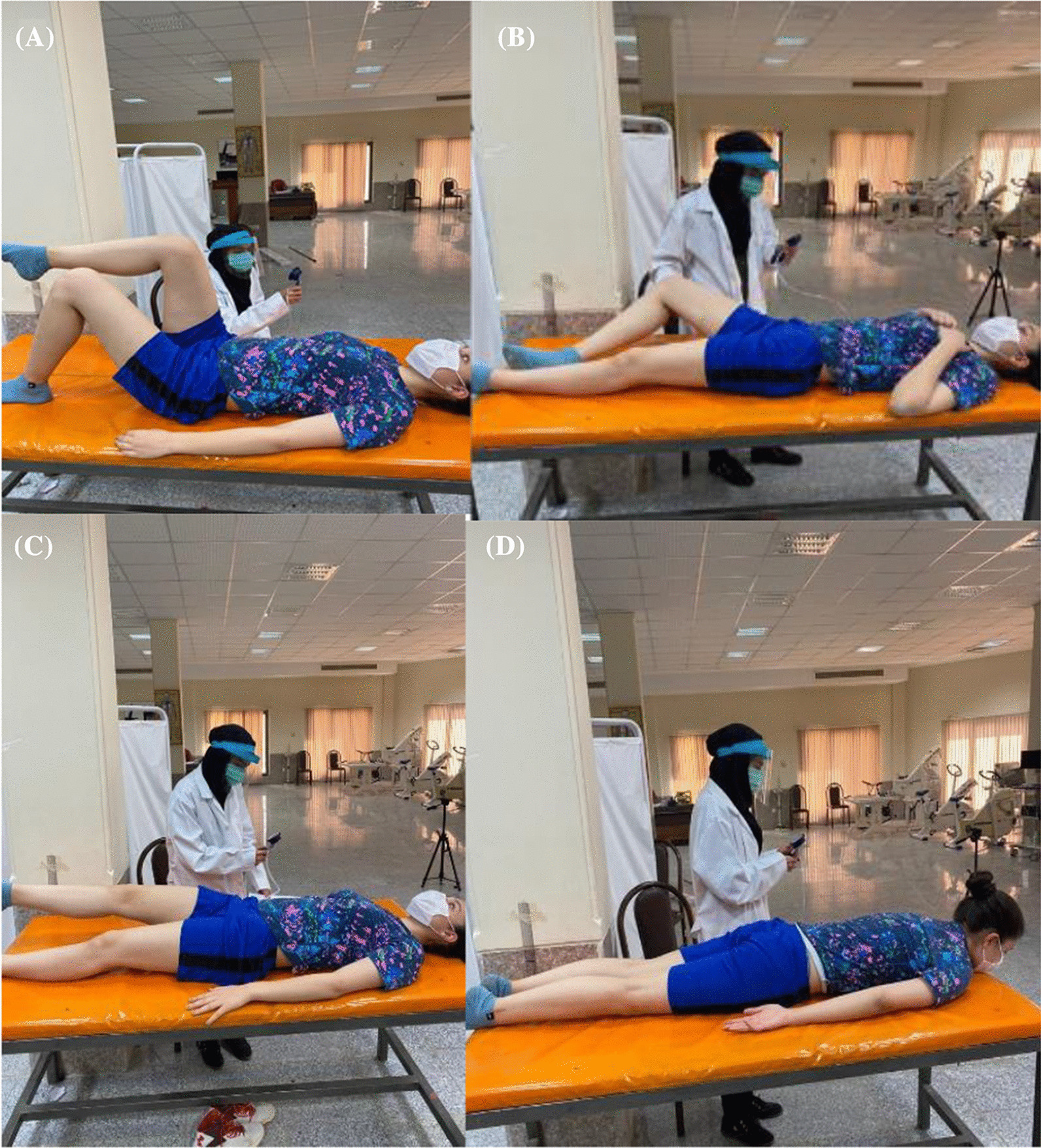
**A** Knee lift abdominal test, **B** bent knee fall-out test, **C** active straight leg raising test, **D** PRONE test

- For the KLAT, the subjects were in a crook lying position, and the pressure biofeedback unit (PBU) was placed horizontally under the spine of the participants, with the lower edge at the level of the posterior superior iliac spines (PSIS); the basal pressure of the PBU was inflated to 40 mmHg (baseline pressure). The participants were asked to lift one foot off the examination table until reaching the hip and knee flexion of 90° and hold for 4–6 s. Following that, the maximal pressure deviation was recorded and used for further analysis (Fig. 3A).
- For the BKFO test, the subjects were supine position, and the PBU was placed vertically under the lumbar spine with the lower edge two centimeters caudal of the PSIS on the contralateral side of the flexed knee. In addition, a folded towel was placed near the PBU to keep both sides of the lumbar spine at the same height. The basal pressure of the PBU was adjusted to 40 mmHg (baseline pressure). Afterwards, one hip was flexed, and the knee was also flexed to 120°, with the foot resting on the surface of the examination couoch. The participants were asked to slowly bend their hip to approximately 45° of the abduction/lateral rotation while keeping their foot supported beside their straight knee and return to the starting position. The maximal pressure deviation was recorded and used for further analysis (Fig. 3B).
- The ASLR was also executed in the supine position, and the PBU placed horizontally under the lumbar spine of the participants, with the lower edge at the level of the PSIS; the calf of PBU was adjusted to 40 mmHg (baseline pressure). The participants lifted one extended leg 20 cm above the examination bed and held it for 20 s. The maximal pressure deviation was also recorded for further analysis (Fig. 3C).
- In the PRONE test, the participants were positioned prone on the examination table. The PBU was placed between the anterior superior iliac spine and navel and inflated to 70 mmHg (baseline pressure). Afterwards, the subjects were asked to breathe deeply using their abdominal wall. After the completion of two normal breaths, the inflatable bag was readjusted to 70 mmHg. The subjects were requested to perform three contractions with the following verbal command: “Draw in your abdomen without moving your lumbar spine or pelvis and hold the position until you are told otherwise.” Using palpation, the examiner determined whether the participants were moving their spine/pelvis over 10 s (Fig. 3D).

To determine the LPC status of the participants in the groups with proper and poor LPC, the pressure changed from the baseline pressure (40 mmHg for supine tests or 70 mmHg for prone test), and the values of each test were recorded by the researcher. If the mean pressure change of all the tests was >|± 8| mmHg, the poor LPC group would be considered. If the mean pressure change of all the tests was ≤|± 8| mmHg, proper LPC would be considered. Table 1 shows the mean values of the four LPC tests in the study groups [15, 23, 29].

**Table 1:** Descriptive statistics of participants with proper LPC and poor LPC

	Poor LPC (n = 17)		Proper LPC (n = 17)		*P*-value
	Mean	SD	Mean	SD	
Age (years)	18.29	2.41	18.29	4.10	1.00
Weight (kg)	67.76	16.79	65.82	9.25	0.62
Height (cm)	173.23	8.26	173.76	6.24	0.84
BMI (kg m^−2^)	22.33	4.13	21.72	2.47	0.66
Activity (h wk^−1^)	5.27	1.92	5.55	2.14	0.67
Duration of activity (years)	7.47	2.42	7.17	1.81	0.79
KLAT (mmHg)	10.17	2.65	3.76	2.99	0.0001
BKFO (mmHg)	14.47	2.85	2.94	2.13	0.0001
ASLR (mmHg)	10.88	2.28	3.76	2.99	0.0001
PRONE (mmHg)	17.76	3.88	6.35	3.25	0.0001

*LPC* lumbopelvic control, *SD* standard deviation, *BMI* body mass index, *h* hours, *wk* week, *KLAT* knee lift abdominal test, *BKFO* bent knee fall out, *ASLR* active straight leg raising

### Statistical analysis

Data analysis was performed using SPSS version 21.0 (SPSS Inc., Chicago, USA) employing the Shapiro–Wilk test to assess the normality of data distribution and Levene’s test for the homogeneity of data. In addition, descriptive statistics were calculated for all variables, and mean and standard deviation (SD) and 95% confidence interval (CI) were reported. The homogeneity of the demographic data at baseline was evaluated using independent samples t-test, which was also employed to compare the means of the landing mechanics and muscle EMG variables between the participants with proper and poor LPC. In all the statistical analyses, the significance level was set at 0.05.

The sample size of the study was estimated at 17 participants per group based on the study by Grosdent et al. [15], and the effect size (ES) was calculated using the following formula:

$$ES = \frac{{t^{2} }}{{t^{2} + \left( {n1 + n2 - 2} \right)}}$$

ES with *d* < 0.2 was considered to be small, while *d* > 0.5 was moderate, and *d* > 0.8 was considered large [30].

## Results

In total, 34 female basketballs, volleyball, and handball players completed the study. The results of the Shapiro-Wilks and Levene’s tests confirmed that the data were normally distributed, and the variances were homogeneous (*P* > 0.05). In addition, the comparison of the participants with proper and poor LPC indicated no significant differences regarding age, weight, height, body mass index, activity hours in the week, and duration of activity (*P* > 0.05) (Table 1).

### Muscle activity

**Fig. 4:**
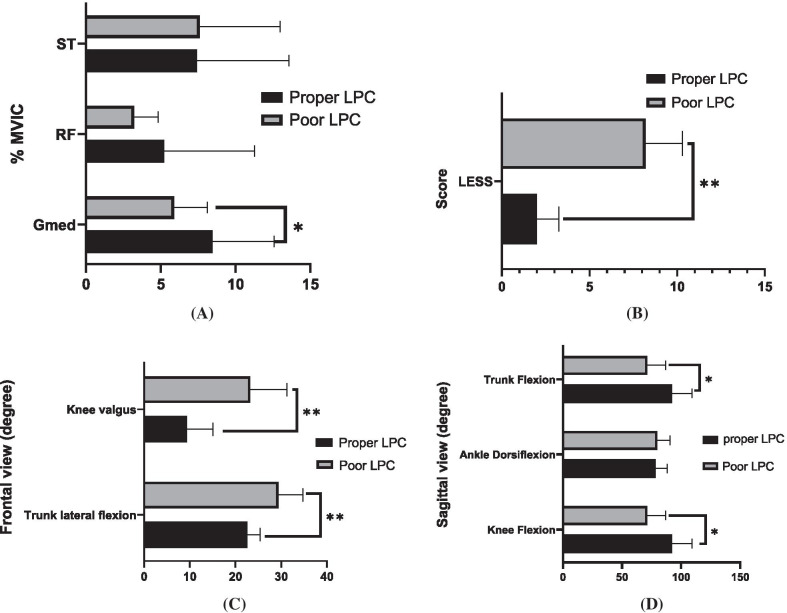
Comparison of study variables (Mean ± SD): **A** muscles activity (% MVIC); **B** landing error scoring system (LESS); **C** landing mechanics/frontal view; **D** landing mechanics/Sagittal view, between subjects with proper and poor LPC (*P < 0.05; **P < 0.001, respectively; error bars indicate standard deviations)

A difference was observed in the GMed activity between the study groups (t_32_ = 2.26; 95% CI 0.26–4.89; *P* = 0.032; ES = 0.13). However, no significant differences were observed between the groups in terms of the RF and ST muscle activity (*P* > 0.05) (Table 2 and Fig. 4A).

**Table 2:** Comparison of muscles activity during single-leg standing and landing mechanics between participants with proper LPC and poor LPC

	Poor LPC (n = 17)		Proper LPC (n = 17)		*P*-value	ES
	Mean	SD	Mean	SD		
Electromyography* (%*MVIC*)						
GMed	5.89	2.22	8.47	4.12	0.032*	0.13
RF	3.23	1.52	5.21	6.06	0.20	0.05
ST	7.61	5.36	7.42	6.14	0.92	0.00
*Landing mechanic*						
Trunk lateral flexion (°)	29.40	3.78	22.60	2.77	0.0001**	0.37
Knee abduction (°)	23.26	8.02	9.40	5.70	0.0001**	0.48
Trunk flexion (°)	74.66	11.17	87.40	15.49	0.015*	0.16
Knee flexion (°)	71.40	15.52	92.40	16.70	0.001**	0.28
Ankle dorsiflexion (°)	79.86	10.58	78.26	9.88	0.67	0.00
LESS (errors)	8.20	2.11	2.00	1.25	0.0001**	0.74

*LPC* lumbopelvic control, *SD* standard deviation, *ES* effect size, *GMed* gluteus medius, *RF* rectus femoris, *ST* semitendinosus, *MVIC* maximum voluntary isometric contraction, *LESS* landing error scoring system. *Percent activation relative to MVIC. *P < 0.05; **P < 0.001

### Landing kinematics

The analysis of knee abduction indicated a significant difference between the subjects with proper and poor LPC (t_32_ = − 5.45; 95% CI − 19.07 to 8.66; *P* = 0.0001; ES = 0.48). Furthermore, a significant difference was observed between the subjects with proper and poor LPC regarding the lateral flexion angle of the trunk in the frontal plane (t_32_ = 7.09; 95% CI 5.54–10.05; *P* = 0.0001; ES = 0.61). Significant differences were also denoted between the two groups in terms of the trunk flexion angle (t_32_ = 2.58; 95% CI 2.63–22.83; *P* = 0.015; ES = 0.16) and knee flexion angle in the sagittal plane (t_32_ = 3.56; 95% CI 8.93–33.06; *P* = 0.001; ES = 0.28), while no significant difference was observed in ankle dorsiflexion (*P* > 0.05). In the LESS test, a significant difference was also observed between the subjects with proper and poor LPC (t_32_ = − 9.78; 95% CI − 7.49 to 4.90; *P* = 0.0001; ES = 0.74) (Table 2 and Fig. 4B–D).

## Discussion

The present study evaluated the status of LPC in professional female athletes engaged in sports requiring frequent jump-landing, and compared the mechanical status of the landing and lower limb muscle activity of the athletes with proper and poor LPC to try and identify the associated risk factors. Lumbopelvic control was assessed by four tests using a PBU in which mean pressure changes of more than |± 8| mmHg indicated poor LPC. Based on these results, the athletes participating in the study were classified as poor LPC and proper LPC.

The current study indicated that professional female basketball, volleyball, and handball players have poor LPC. The lumbopelvic region provides dynamic stability for the movement of the extremities. The risk of injury increases with the disruption of the elements within the kinetic chain, which in turn causes alterations in the biomechanics of the extremities [31]. The trunk muscle activity precedes lower limb muscle activity, and the central nervous system provides a stable foundation for lower limb movements [32]. Therefore, the disrupted stability of this foundation could lead to inefficient landing and predispose the athlete to lower limb injury. Our findings indicated that 50% of knee injuries occurred after landing on the other foot of the athlete, and the other 50% arise from improper landing [4].

According to findings of the current research, landing mechanics (i.e., LESS test scores) were significantly inefficient in the subjects with poor LPC compared to those with proper LPC, which confirms the research hypothesis. This may result from poor control of the lumbopelvic region as weaknesses or delays in the activation of the muscles involved in LPC could cause compensatory and unpredictable movements. For instance, GMed is one of the main lumbopelvic muscles, and the main abductor of the hip joint. Dysfunction of this muscle could cause the femur to move to the midline of the body and increase the dynamic knee valgus moment [33]. A LESS test score greater than 5 increases the relative risk of ACL injury 10.7 times. On the other hand, core exercises could reduce the LESS score by up to three points [34]. In other words, the increased stability of the trunk could decrease the mechanical errors of landing, which is consistent with our findings. Also, impaired neuromuscular control of core stability during dynamic movements may expose athletes to lower limb injuries [22].

According to the results of the present study, lateral flexion of the trunk and knee abduction were significantly higher in the subjects with poor LPC compared to those with proper control.

In the frontal plane, landing imposes on the knee high strains on the medial collateral ligament and ACL. Furthermore, maintaining and controlling the trunk plays a key role in reducing the pressure on the knees, and the risk of lower limb injuries. In particular, the combination of knee valgus and lateral trunk flexion on the frontal plane is highly sensitive in the identification of women at risk of ACL injuries [35]. In this regard, individuals with lateral trunk flexion have increased knee valgus, and lateral flexion of the trunk could cause the ground reaction force to pass through the lateral compartment of the knee and increase the abduction torque exerted on it [9]. Previous research reported that the neuromuscular control of the trunk and knee predicts the risk of anterior cruciate ligament injury with high sensitivity and specificity [22, 36, 37], in line with the results of the present study. Therefore, LPC should be considered a sensitive predictor of lower limb injuries in athletes engaged in sport requiring frequent landing.

According to the results of the present study, flexion of the knee and trunk at the sagittal plane was higher in the subjects with proper LPC compared to those with poor LPC, which confirmed the research hypothesis. Knee flexion angle has been associated with knee injuries, with several studies reporting that and increased knee flexion angle could decrease the anterior shear force on the knee joint and strain the ACL [17, 38, 39]. In this regard, with the knee flexed, lower anterior shear forces are applied to the knee joint compared to the extension position: trunk flexion with knee flexion at the moment of landing could cause decrease the ACL strain [40]. Furthermore, decreased LPC stability is associated with decreased sagittal plane motion during single-leg squatting, as well as increased frontal plane motion during single-leg drop vertical jumping [26].

The activity of three important muscles investigated in the current research were RF, ST, and GMed, which originate from the pelvis and are involved in the occurrence of knee injuries [9, 31, 41, 42]. According to our findings, only the GMed muscle activity was significantly different between the subjects with proper and poor LPC regarding the ability to stand on one leg. During weight bearing, the GMed controls the pelvis and the femur in the frontal plane. Clinicians and researchers often emphasize the importance of strengthening the hip musculature to stabilise the pelvis and decrease valgus alignment at the knee level [43]. In fact, GMed plays a vital role in LPC, and, given its abduction torque, it influences the mechanics of the lower limb, especially knee valgus. This result is in line with previous findings, which demonstrated that the GMed muscle is a primary pelvic stabilizer during single-legged standing and also plays a key role in LPC [44]. In this regard, our findings are also consistent with the study by Kim et al., which indicated that the level of GMed activity is more important for controlling knee and pelvic stability in the frontal plane as opposed to the onset of activation [45], again in line with the current research.

One of the limitations of our study was that, given our Islamic country sharia laws, it was not possible to evaluate men and women, and the women evaluator was only allowed to study women. Given the lack of a force plate, we could not measure the activity of the studied muscles at the moment of foot contact to the ground while landing. Moreover, it was assumed that the LPC tests used in our study are an accurate representation of LPC. Therefore, it is recommended that further investigations be focused on the effects of the lower extremity muscle EMG on the landing task and more muscles to achieve more accurate results and make comparisons with our findings. Also, as we measured EMG during one-leg standing, careful interpretation and implication of the findings should be performed to a real situation or sports tasks.

## Conclusion

According to the results of the present investigation, poor LPC in the professional athletes engaged in sport requiring frequent jump-landings increased the scores of the mechanical landing test (LESS), as well as the lateral flexion angle of the trunk, and angle of dynamic knee abduction, while decreasing the knee flexion angle, flexion angle of the trunk, and GMed activity. Therefore, athletes with poor LPC may experience biomechanically improper landing, which increases the risk of lower extremity injury, especially knee injuries. Our findings highlight the need for LPC measurement in the evaluation of injury risk factors, and further investigations are required regarding the impact of LPC training on the risk of injuries.

## Data Availability

The datasets used and/or analysed during the current study are available from the corresponding author on reasonable request.

## Abbreviations

ACL: Anterior cruciate ligament
KLAT: Knee lift abdominal test
BKFO: Bent knee fall-out
ASLR: Active straight leg raising
PBU: Pressure biofeedback unit
LESS: Landing error scoring system
EMG: Electromyography
GMed: Gluteus medius
RF: Rectus femoris
ST: Semitendinosus
PSIS: Posterior superior iliac spines
ASIS: Anterior superior iliac spine
ICC: Intraclass correlation-coefficient
SD: Standard deviation
CI: Confidence interval
ES: Effect size
