# Correction to: Effect of lumbopelvic control on landing mechanics and lower extremity muscles’ activities in female professional athletes: implications for injury prevention

**DOI:** 10.1186/s13102-021-00339-4

**Published:** 2021-09-09

**Authors:** Paria Fadaei Dehcheshmeh, Farzaneh Gandomi, Nicola Mafulli

**Affiliations:** 1 Department of Sport Injuries and Corrective Exercises, Sports Sciences Faculty, Razi University, Kermanshah, Iran; 2 Department of Musculoskeletal Disorders, Via Salvador Allende, 43, 84081 Baronissi, Italy; 3 Clinica Ortopedica, Ospedale San Giovanni di Dio E Ruggi D’Aragona, Largo Città di Ippocrate, 84131 Salerno, Italy; 4 Barts and the London School of Medicine and Dentistry, Centre for Sports and Exercise Medicine, Mile End Hospital, Queen Mary University of London, 275 Bancroft Road, London, E14DG England; 5 School of Pharmacy and Bioengineering, Faculty of Medicine, Guy Hilton Research Centre, Keele University, Thornburrow Drive, Hartshill, Stoke-on-Trent, ST4 7QB England

## Correction to: BMC Sports Sci Med Rehabil (2021) 13:101 https://doi.org/10.1186/s13102-021-00331-y

Following publication of the original article [1], the authors identified an error in the author name of Paria Fadaei Dehcheshmeh.

The incorrect author name is: Paria Fadari Dehcheshmeh.

The correct author name is: Paria Fadaei Dehcheshmeh.

The author group has been updated above and the original article [1] has been corrected.
